# Association of Self-Perceived Psychological Stress with the Periodontal Health of Socially Deprived Women in Shelter Homes

**DOI:** 10.3390/ijerph18105160

**Published:** 2021-05-13

**Authors:** Syeda A. Tanveer, Ashar Afaq, Montaser N. Alqutub, Nada Aldahiyan, Abdulrahman M. AlMubarak, Amynah C. Shaikh, Mustafa Naseem, Fahim Vohra, Tariq Abduljabbar

**Affiliations:** 1 Department of Oral Biology, College of Dentistry, Dow International Dental College, Karachi 74200, Pakistan; abeerah.tanveer@duhs.edu.pk (S.A.T.); amynah.tariq@duhs.edu.pk (A.C.S.); 2 Department of Community and Preventive Dental Sciences, College of Dentistry, Dow International Dental College, Karachi 74200, Pakistan; ashar.afaq@duhs.edu.pk (A.A.); mustafa.naseem@duhs.edu.pk (M.N.); 3 Department of Periodontics and Community Dentistry, College of Dentistry, King Saud University, Riyadh 11545, Saudi Arabia; alqutub@hotmail.com (M.N.A.); amalmubarak@ksu.edu.sa (A.M.A.); 4 Department of Restorative Dentistry, Division of Operative Dentistry, King Saud University, Riyadh 11545, Saudi Arabia; naldahiyan@ksu.edu.sa; 5 Department of Prosthetic Dental Science, College of Dentistry, King Saud University, Riyadh 11545, Saudi Arabia; fvohra@ksu.edu.sa

**Keywords:** periodontitis, chronic stress, psychological stress, negative life events

## Abstract

The present study aimed to assess the effect of self-perceived psychological stress on the periodontal health of socially deprived women. The study included three hundred and eighty-five socially deprived women residing in shelter homes. The presence of stress and its severity was assessed by using Sheldon Cohen’s 10-item perceived stress scale (PSS), and periodontal health status was assessed utilizing the community periodontal index. Statistical analyses were performed using an independent sample t-test, a one-way ANOVA, the Pearson chi-Square test, and binary logistic regression. *Results:* A total of 385 samples were included, the majority of whom (*n* = 297; 72.5%) belonged to the age group of 15–30 years. There were 34 (8.8%) participants who were educated up to graduate level. A total of 47.8% of the women were found with healthy periodontal status, and 52.5% of the samples were diagnosed with major psychological stress. Half of the samples (201-52.2%) had a periodontal problem. The mean PSS was found statistically significant concerning age group, education, and psychological stress level. In the univariate logistic regression analysis, a significant association of periodontal status was observed with the age group 31–45 years [(OR = 1.76; 95% C.I (1.11–2.78)] and with a major psychological stress level [(OR = 2.60; 95% C.I (1.72–3.93)]. Psychosocial stress among socially deprived women was found to be a risk factor for periodontal disease.

## 1. Introduction

Periodontitis is defined as the inflammation and destruction of the supporting and surrounding structure of teeth. It originates from the gingival tissue and is characterized by inflammation (redness, swelling, pain, loss of function), accompanied by spontaneous or induced gingival bleeding and altered bone homeostasis, causing tooth mobility and tooth loss [1]. Periodontitis has a multifactorial etiology, and the cumulative effect of genetics, environmental factors, oral/systemic health status, and social habits result in the variation of the host immune response in the periodontal disease process [2,3]. Both local factors (including dental crowding, misalignment, and oral hygiene practices), and systemic factors (including alteration in gingival circulation, salivary flow rate, serum blood sugar level, and hormonal disturbance), might influence the host immune response [4,5].

Stress is defined as a state of disharmony in body homeostasis, counteracted by an intricate repertoire of physiologic and behavioral responses that aim to maintain and re-establish the threatened homeostasis (adaptive stress response) [6]. Psychological stress triggers the neuro-endocrine system, resulting in the continued release of pro-inflammatory mediators and an alteration in normal microbiota, thereby leading to periodontal inflammation [7,8,9]. The effect of different aspects of psychological stress, including emotional stress, work-related stress, and dissatisfaction at home or in society, is associated with compromised oral health as suggested by previous studies [10,11]. In previous literature by Pistorius et al. and Wimmer et al., a correlation has been found between increased level of chronic stress and periodontitis with clinical attachment loss [8,9].

Periodontitis itself is considered as a predisposing factor for various systemic diseases such as atherosclerotic heart disease, stroke, diabetes mellitus, or pre-term low birth weight [5]. Studies have shown that women, as compared to men, are more likely to develop chronic stress due to the detrimental influence of minor daily life stresses experienced over a lifetime. The gender differences in stress coping strategies predicted that men use more active and instrumental coping behaviors, and women use more passive and emotion-focused behaviors [12,13]. Among available literature on the effect of stress on periodontal health, negligible evidence exists for socially deprived women combating stressful life in shelter homes. In a study by Wickham et al., the impact of social deprivation was found to be associated with an increased risk of stress and depression among women [14]. It was hypothesized that increased psychological stress levels among socially deprived women would show a clinically compromising influence on their periodontal health. Therefore, this study aimed to assess the effect of self-perceived psychological stress on the periodontal health of socially deprived women.

## 2. Materials and Methods

### 2.1. Study Settings and Participants

The present cross-sectional study included socially deprived women residing in four different shelter homes located in Karachi, Pakistan. The study was conducted from December 2019 to March 2020. Shelter homes were categorized as Shelter A, Shelter B, Shelter C, and Shelter D. A minimum sample of 384 was calculated, with a 95% confidence interval, 5% level of significance, and 5% margin of error. The sample size was increased to 410 by adding 5% in order to overcome loss to follow-up. The sample size was calculated via source available at https://www.surveysystem.com/sscalc.htm (accessed on 31 December 2020). The total population size of women in Karachi was recorded at 5,637,746 (https://www.worldometers.info/world-population/pakistan-population, accessed on 31 December 2020). Women residents of shelter homes with an age range of 15–45 years were included in the study. Women who did not give consent or were pregnant, had diabetes mellitus, dementia, and hypodontia/anodontia were excluded. A convenience sample approach was adopted to include women.

### 2.2. Ethical Considerations 

The study protocol was developed in line with the ethical principles of the declaration of Helsinki (amended in 2013). The DUHS Institutional Review Board reviewed and approved the research protocol of the study (Ref: IRB-1592/DUHS/Approval/2020/91). The administration of respective shelter homes granted the permission for performing the investigation. The study’s subjects were presented with informed consent forms for their voluntary participation. Participating women could withdraw from the study without any consequences.

### 2.3. Study Questionnaire

A self-administered questionnaire was created in English and translated into the Urdu language for the convenience of the participating women [15]. It comprised of two sections. The first section had questions about socio-demographic factors, and Sheldon Cohen’s 10-itemperceived stress scale (PSS) based on a 5-point Likert scale (0 = never, 1 = rarely, 2 = occasionally, 3 = often, 4 = always) [16]. The statistician, along with the authors, reviewed the content of each question to make sure that the survey reflected appropriate phrasing, understanding, and validation. A pilot study was performed by distributing the questionnaire to 30 subjects, followed by a principal component analysis to validate the questionnaire. After the response from the participating women, the questionnaire was again translated to English for assessment. The consistency of the questionnaire was assessed by Cronbach’s alpha (0.70). The cumulative score of the PSS questions was categorized as “minor stress” (PSS score < 20/40) and “major stress” (PSS score > 20/40). The reference of cut-off used was adopted from the work of Pizzagali et al. [17].

The second section was composed of the Community Periodontal Index (CPI) employed for a periodontal health examination. The identities of the women participants were not recorded throughout the data collection process to ensure confidentiality. Once all the women had given verbal and written informed consent, they were requested to fill in a self-reported questionnaire and to undergo periodontal examinations as part of a dental health check-up.

### 2.4. Clinical Investigations

For the periodontal examination, a CPI periodontal probe was utilized. The probe was walked around, measuring the depth of the gingival crevices and periodontal pockets with a force of approximately 20–25 gram force by a single operator MN to maintain homogeneity. Only the permanent central incisors and the permanent first and second molars of both the maxilla and the mandible were recorded; these were 16/17, 11, 26/27 for the maxillary arch and 46/47, 31, 36/37 for the mandibular arch. The coding criteria was: 0 = healthy, 1 = bleeding, 2 = calculus, 3 = pocket depth of 4–5mm (black band on probe is partially visible), 4 = pocket depth of 6mm or more (black band on probe is not visible), X = excluded sextant, and 9 = not recorded [18]. 

The education variable was coded as: 0 = uneducated, 1 = undergraduate (less than 10 years of education), 2 = graduate (more than 10 years of education); age group was coded as: 1 = 15–30 years, 2 = 31–45 years; periodontal status was coded as: 1 = healthy, 2 = diseased; stress was coded as: 1 = major stress, 2 = minor stress. Fleiss’ kappa was run to determine if there was agreement among the examiner’s judgment on the CPI score of typodont on manikins (PER 1001-UL-SP-FEM-32). Six non-unique dental interns were chosen at random from a group of 50 dental interns to perform the examination. Each examiner rated in a separate room so they could not influence the decision of other examiners. Fleiss’ kappa showed that there was good agreement among the operators’ judgments: *κ* = 0.725 (95% CI, 0.720 to 0.729), *p* < 0005.

### 2.5. Statistical Data Analysis

The data were stored and analyzed using statistical software (IBM-SPSS version 23.0, IBM, Armonk, NY, USA). Counts with percentages were given for qualitative parameters such as age group, education of women, and psychological stress level; means with standard deviation were given for quantitative parameters, including age in years and perceived stress scores (PSS). Cronbach’s alpha was used to test the reliability of perceived stress scale items. We used an independent sample t-test to compare the mean PSS with age group, periodontal status, and psychological stress level; a one way ANOVA was used to compare the mean PSS with education levels. The Pearson chi-Square test was performed to assess the association of periodontal status with age group, education, and psychological stress. A binary logistic regression analysis with studied parameters was performed to estimate the risk of periodontal disease, and an odds ratio with a 95% confidence interval was reported for univariate and multivariate models. The reported *p*-values of less than 0.05 were considered statistically significant.

## 3. Results

A total of 410 women were included in the present study. There were 400 women who consented to take part, and 385 were found to be eligible based on the inclusion and exclusion criterion, attaining a response rate of 93.3%. Table 1 reports the baseline characteristics of the studied women. In the present study, there were 385 samples, among which 72.5% were found in the age group of 15–30 years. From among all the subjects, 77.1% reached the graduate level of education, while 47.8% of the women were found with healthy periodontal status. A total of 52.5% of the samples were diagnosed with major psychological stress. 

Results of an independent sample *t*-test showed women from the age group 15–30 years with larger mean values for PSS as compared to women 31–45 years old. When we compared the mean values of PSS with education levels, psychological stress results showed that the mean difference was statistically significant (*p* < 0.05).

The descriptive statistics of the periodontal status of women by age, education, and psychological stress are presented in Table 2. Among periodontally diseased women, 67.2% belonged to the age group 15–30 years old. Likewise, 70.1% had undergraduate education, and 63.7% were found with major stress. The Pearson chi-Square test showed a significant difference in periodontal status by age group, education, and psychological stress (*p* < 0.05).

Table 3 demonstrates the results of binary logistic regression in the univariate analysis. It was observed that age and psychological stress had a significant positive association with periodontal disease, with participants aged 31–45 years 1.76 times more likely to be found with periodontal disease; moreover, women participants with major stress are more than twice likely to be found with periodontal disease as compared to other women participants. However, in a multivariate model, age and psychological stress showed significant association with periodontal status (*p* < 0.05).

## 4. Discussion

In the present study, the number of women with major stress (*n* = 202 (52.5%)) was higher as compared to women with minor stress (*n* = 183 (47.5%)). Also, the diseased periodontal status was found to be higher in women participants with major stress as compared to women with minor stress (*p* < 0.001). In addition, in the present study, a statistically significant relationship was observed between diseased periodontal status and individuals among the age group 31–45 years, in both the univariate and multivariate analyses (*p* < 0.001, OR = 1.76, *p* < 0.05. OR 0.02). Several studies have shown that the prevalence and severity of periodontitis increase with age, and chronic periodontal diseases with bone loss occur most frequently in individuals under the age of 50 [19]. Periodontal disease has a reciprocal relationship with the level of education. It is reported that the higher the level of education, the lower the chances of developing periodontal disease [20,21]. The regression model of the univariate analysis in this study showed a statistically insignificant relation between periodontal status and education (*p* > 0.05).

The psycho-physiological response of a living organism to a perceived challenge or threat is referred to as ‘stress’ [22]. Stress can cause a person to neglect oral hygiene, with a resultant unfavorable effect on the periodontal tissues. The association of stress with periodontal health is difficult to prove as many factors influence the incidence and severity of periodontitis. Negative life events among socially deprived women can be linked with the onset or exacerbation of illness, and the relationship between important negative life events and disease can be mediated by the immune system [7]. Research has shown that emotional stress can modulate the immune system through the neural and endocrine systems [23].

In the present study, the diseased periodontal status was found to be more inclined towards participants with major stress. Moreover, a significant relationship was observed between self-perceived psychological stress and periodontal disease. Coelho et al. and Moss et al. found significant positive associations between stress and periodontal pocket depth, stress and clinical attachment loss, and stress and periodontitis [24,25]. Croucher et al. showed that periodontitis was associated with the detrimental impact (*p* < 0.01) and frequency *(p* < 0.05) of negative life events and unemployment (*p*< 0.05); notably, these associations remained statistically significant after adjusting oral health behavior and socio-demographic variables [26]. Moreover, Refulio et al. showed a significant association of stress and depression in terms of salivary cortisol levels with chronic periodontitis [27]. Thus, women with chronic periodontitis had significantly higher levels of salivary cortisol than women without chronic periodontitis. Chiou, in a study utilizing the community periodontal index, showed that the married and divorced/widowed women were at greater risk of having poorer periodontal health as compared to solitary women [28]. In our study, marital status and its association with periodontitis was not assessed and this can be considered as one of the possible limitations.

One plausible hypothesis for the above findings is that the activated neuro-endocrine (hypothalamic-pituitary-adrenal) system in anxiety and depression results in a continued release of glucocorticoids, thereby leading to inflammation [8]. Furthermore, in emotional disorder, the patients’ sympathetic nervous system releases adrenaline and nor-adrenaline from the adrenal medulla, which exert an immunosuppressive effect [7]. A recent systematic review by Castro et al. reported the role of cortisol, the glucocorticoid stress biomarker, suggesting that its elevated production causes detrimental effects on the immune responses [28]. In addition, alterations in the release of inflammatory cytokines especially elevate the levels of IL-B1 (interleukin-Beta 1)—which deregulate the host immune response and alter normal microbiota—consequently exacerbating chronic conditions such as periodontitis [29]. Deinzer et al. recognized that microorganisms possess the ability to recognize hormones within the host and utilize them to adapt to their surroundings [30]. This supports the theory that psychological stress may favor the development of many bacterial infections. In a study by George M Slavich and Julia Sacher, it was concluded that sex hormones have synergistic effects on immune system activity. Particularly, female sex hormones (estrogens) are associated with pro-inflammatory function at different concentrations [31]. Moreover, fluctuations in sex hormones influence the response of individuals towards negative life events over time.

On the contrary, some studies have suggested minimal to no association between stress and periodontal health [32]. This could be due to the lack of a standard scale, and variability in the selection of different instruments and scales utilized for psychological assessment. Furthermore, there are biological markers or diagnostic tools that could safely detect most psychiatric disturbances [33]. The psychological variables are usually measured by self-reported scales and do not allow an assessment of the subjective and behavioral aspects of individuals. One should bear in mind that the participant’s validity and reliability may vary when analyzing internal consistency and sensitivity to external factors, which are known to influence their decision [34]. Stress is not experienced in the same manner by everyone; the experience is subjective and it depends on how much support is being provided to the individual by family and friends. Such support could lessen the physical and psychological impact of stress. Furthermore, how a person manages stress is more important than the presence of stress factors [9]. In addition, the clinical parameters and the use of different indices, e.g., gingival index (GI), plaque index (PI), clinical attachment loss (CAL), and periodontal disease index (PDI) in different studies can produce variation in the assessment of periodontal status. 

Not using a non-multicentric study design for the assessment of the subjective and behavioral aspects of individuals can be responsible for the possible limitations of the present study. Moreover, the results of the present study can only be applicable to a cohort of socially deprived women in shelter homes and not all women. It is pertinent to mention that the influence of stress on periodontal disease cannot be neglected, as many studies have reported the role of stress in periodontal disease [8,9]. Hence, taking into consideration the contradictory findings of various studies, future studies might find it beneficial to evaluate the differences in the molecular pathways of stress among socially deprived and stressed women to determine the exact relationship between stress and periodontal disease. Furthermore, it is critical to identify the role of sex hormones and biomarkers that are useful in affecting periodontal health, which will aid in deciphering the relationship between stress and periodontitis among older women. These findings could contribute immensely to our understanding of the role of stress in periodontitis, and offer avenues for intervention.

## 5. Conclusions

The study showed a significant association between psychosocial stress and periodontitis among socially deprived women residing in shelter homes. Regression analysis showed an increased probability of developing periodontitis in women suffering from psychosocial stress. These results recommend that a multidisciplinary approach (dentist, psychologist/psychiatrist, and neurologist) should be utilized in the identification of patients with social stress, and to adopt counter-measures to overcome its harmful effects on periodontal health among women.

## Figures and Tables

**Table 1:** Mean comparison of PPS with baseline characteristics (*n* = 385).

Parameters		Total	Perceived Stress Scores (PSS)	
		n (%)	Mean (SD)	*p*
Age group	15–30	279 (72.5)	15.3 (6.2)	0.045 *
	31–45	106 (27.5)	13.6 (7.9)	
	Mean (±SD)	25.2	SD = ± 9.7	
Education	Uneducated	54 (14.0)	13.6 (6)	0.047 *
	Undergraduate	297 (77.1)	15.3 (6.9)	
	Graduate	34 (8.8)	12.9 (5.5)	
Periodontal status	Healthy	184 (47.8)	14.3 (5.4)	0.12
	Diseased	201 (52.2)	15.4 (7.7)	
Psychological stress	Major stressor	202 (52.5)	16.5 (7.2)	<0.01 *
	Minor stressor	183 (47.5)	13 (5.6)	
Reliability Coefficient	Cronbach’s α	10 items	0.80	

* denotes presence of statistical significance.

**Table 2:** Periodontal status by age group, education, and psychological stress.

Characteristics		Periodontal Status		*p*
		Healthy (*n* = 184)	Diseased (*n* = 201)	
		*n* (%)	*n* (%)	
Age Group	15–30	144 (78.3)	135 (67.2)	0.015 *
	31–45	40 (21.7)	66 (32.8)	
Education	Uneducated	16 (8.7)	38 (18.9)	<0.01 *
	Undergraduate	156 (84.8)	141 (70.1)	
	Graduate	12 (6.5)	22 (10.9)	
Psychological stress	Major stressor	74 (40.2)	128 (63.7)	<0.01 *
	Minor stressor	110 (59.8)	73 (36.3)	

* denotes presence of statistical significance.

**Table 3:** Risk estimation of periodontal disease with studied parameters.

Parameters		Periodontal Status	
		Univariate OR (95% C.I)	Multivariate ^a^ OR (95% C.I)
Age Group	15–30	Ref.	Ref.
	31–45	1.76 * (1.11–2.78)	0.02 * (0.0–0.07)
Education	Graduate	Ref.	Ref.
	Uneducated	1.29 (0.51–3.23)	1.82(0.65–5.11)
	Undergraduate	0.49 (0.23–1.03)	1.15(0.48–2.77)
Psychological stress	Minor stressor	Ref.	Ref.
	Major stressor	2.60 * (1.72–3.93)	2.43(1.50–3.94)

* Odds ratio considered significant *p* < 0.05. ^a^ Models were adjusted for all variables in the table.

## Data Availability

The data of the study presented is available from the authors of the article on individual request.

## References

[1] Javed F., Abduljabbar T., Vohra F., Malmstrom H., Rahman I., Romanos G.E. (2017). Comparison of periodontal parameters and self-perceived oral symptoms among cigarette smokers, individuals vaping electronic cigarettes, and never-smokers. J. Periodontol..

[2] Mark Bartold P., Van Dyke T.E. (2013). Periodontitis: A host-mediated disruption of microbial homeostasis. Unlearning learned concepts. Periodontol 2000.

[3] Diehl S.R., Wu T., Burmeister J.A., Califano J.V., Brooks C.N., Tew J.G., Schenkein H.A. (2003). Evidence of a substantial genetic basis for IgG2 levels in families with aggressive periodontitis. J. Dent. Res..

[4] Al-Sowygh Z.H., Ghani S.M., Sergis K., Vohra F., Akram Z. (2018). Peri-implant conditions and levels of advanced glycation end products among patients with different glycemic control. Clin. Implant Dent. Relat. Res..

[5] Ameet M.M., Avneesh H.T., Babita R.P., Pramod P.M. (2013). The relationship between periodontitis and systemic diseases-Hype or hope?. J. Clin. Diagn. Res..

[6] Chrousos G.P. (2009). Stress and disorders of the stress system. Nat. Rev. Endocrinol..

[7] Gunepin M., Derache F., Trousselard M., Salsou B., Risso J.J. (2018). Impact of chronic stress on periodontal health. J. Oral Med. Oral Surg..

[8] Pistorius A., Krahwinkel T., Willershausen B., Boekstegen C. (2002). Relationship between stress factors and periodontal disease. Eur. J. Med. Res..

[9] Wimmer G., Janda M., Wieselmann-Penkner K., Jakse N., Polansky R., Pertl C. (2002). Coping With Stress: Its Influence on Periodontal Disease. J. Periodontol..

[10] Vasiliou A., Shankardass K., Nisenbaum R., Quiñonez C. (2016). Current stress and poor oral health. BMC Oral Health.

[11] Settineri S., Rizzo A., Liotta M., Mento C. (2017). Clinical Psychology of Oral Health: The Link Between Teeth and Emotions. SAGE Open.

[12] Matud M.P. (2004). Gender differences in stress and coping styles. Pers. Individ. Dif..

[13] Ptacek J.T., Smith R.E., Zanas J. (1992). Gender, Appraisal, and Coping: A Longitudinal Analysis. J. Pers..

[14] Wickham S., Taylor P., Shevlin M., Bentall R.P. (2014). The impact of social deprivation on paranoia, hallucinations, mania and depression: The role of discrimination social support, stress and trust. PLoS ONE.

[15] Lu W., Bian Q., Wang W., Wu X., Wang Z., Zhao M. (2017). Chinese version of the Perceived Stress Scale-10: A psychometric study in Chinese university students. PLoS ONE.

[16] Cohen S., Kamarck T., Mermelstein R. (1983). A global measure of perceived stress. J. Health Soc. Behav..

[17] Pizzagalli D.A., Bogdan R., Ratner K.G., Jahn A.L. (2007). Increased perceived stress is associated with blunted hedonic capacity: Potential implications for depression research. Behav. Res. Ther..

[18] Barmes D. (1994). CPITN--a WHO initiative. Int. Dent. J..

[19] Wu Y., Dong G., Xiao W., Xiao E., Miao F., Syverson A., Missaghian N., Vafa R., Cabrera-Ortega A.A., Rossa C. (2016). Effect of aging on periodontal inflammation, microbial colonization, and disease susceptibility. J. Dent. Res..

[20] Lunau T., Siegrist J., Dragano N., Wahrendorf M. (2015). The Association between Education and Work Stress: Does the Policy Context Matter?. PLoS ONE.

[21] Aljehani Y.A. (2014). Risk factors of periodontal disease: Review of the literature. Int. J. Dent..

[22] Schneiderman N., Ironson G., Siegel S.D. (2005). Stress and health: Psychological, behavioral, and biological determinants. Annu. Rev. Clin. Psychol..

[23] Goyal S., Gupta G., Thomas B., Bhat K., Bhat G. (2013). Stress and periodontal disease: The link and logic!. Ind. Psychiatry J..

[24] Moss M.E., Beck J.D., Kaplan B.H., Offenbacher S., Weintraub J.A., Koch G.G., Genco R.J., Machtei E.E., Tedesco L.A. (1996). Exploratory Case-Control Analysis of Psychosocial Factors and Adult Periodontitis. J. Periodontol..

[25] Coelho J.M.F., Miranda S.S., da Cruz S.S., Trindade S.C., de S. Passos-Soares J., de M.M. Cerqueira E., da Conceição N. Costa M., Figueiredo A.C.M.G., Hintz A.M., Barreto M.L. (2020). Is there association between stress and periodontitis?. Clin. Oral Investig..

[26] Croucher R., Marcenes W.S., Torres M.C.M.B., Hughes F., Sheiham A. (1997). The relationship between life-events and periodontitis A case-control study. J. Clin. Periodontol..

[27] Refulio Z., Rocafuerte M., de la Rosa M., Mendoza G., Chambrone L. (2013). Association among stress, salivary cortisol levels, and chronic periodontitis. J. Periodontal Implant Sci..

[28] Chiou L.J., Yang Y.H., Hung H.C., Tsai C.C., Shieh T.Y., Wu Y.M., Wang W.C., Hsu T.C. (2010). The association of psychosocial factors and smoking with periodontal health in a community population. J. Periodontal Res..

[29] Castro M.M.L., Ferreira R.D.O., Fagundes N.C.F., Almeida A.P.C.P.S.C., Maia L.C., Lima R.R. (2020). Association between Psychological Stress and Periodontitis: A Systematic Review. Eur. J. Dent..

[30] Deinzer R., Kleineidam C., Stiller-Winkler R., Idel H., Bachg D. (2000). Prolonged reduction of salivary immunoglobulin A (sIgA) after a major academic exam. Int. J. Psychophysiol..

[31] Slavich G.M., Sacher J. (2019). Stress, sex hormones, inflammation, and major depressive disorder: Extending Social Signal Transduction Theory of Depression to account for sex differences in mood disorders. Psychopharmacology.

[32] Shende A., Bhatsange A., Waghmare A., Shiggaon L., Mehetre V., Meshram E. (2016). Determining the association between stress and periodontal disease: A pilot study. J. Int. Clin. Dent. Res. Organ..

[33] Cotrena C., Branco L.D., Fonseca R.P. (2018). Adaptation and validation of the melbourne decision making questionnaire to Brazilian Portuguese. Trends Psychiatry Psychother..

[34] Committee on Psychological Testing, Including Validity Testing, for Social Security Administration Disability Determinations, Board on the Health of Select Populations, Institute of Medicine (2015). Psychological Testing in the Service of Disability Determination.

